# Attach importance to effectiveness of the aids sentinel surveillance program: Study on HIV/STD-related behavioral characteristics and influencing factors of HIV infection among male patients attending STD clinics in a coastal region of Zhejiang Province

**DOI:** 10.1097/MD.0000000000046322

**Published:** 2026-05-12

**Authors:** Libo Zhang, Yajun Ding, Dongxian Ye, Chunxia Xu, Yaner Yu, Yingbin Wang, Ludan Xu

**Affiliations:** a Department of the Public Health Care, Beilun Branch of the First Affiliated Hospital, College of Medicine, Zhejiang University , Ningbo, Zhejiang Province, China.

**Keywords:** AIDS, male patients, risk factors, STD clinics, syphilis

## Abstract

Male patients attending sexually transmitted disease (STD) clinics are a key sentinel population for human immunodeficiency virus (HIV) surveillance. This study analyzed the prevalence of HIV, syphilis, and hepatitis C infections, behavioral intervention characteristics, and associated risk factors among male patients attending STD clinics to evaluate the effectiveness of sentinel surveillance and providing reference data for improving acquired immune deficiency syndrome (AIDS) prevention and control measures. From 2018 to 2021, a total of 400 male patients aged 15 years and older, who actively sought care at the dermatology and STD clinic of a sentinel hospital, underwent face-to-face anonymous questionnaire surveys and blood tests for HIV, syphilis, and hepatitis C antibodies. Their results were compared with those of other outpatient attendees during the same period. Between 2018 and 2021, most of the male patients tested for HIV, syphilis, and hepatitis C were local residents, middle-aged adults, and married individuals. The overall awareness rate of AIDS prevention and treatment was 76.18%, with a year-on-year improvement. Among the participants, 42.88% had received condom promotion and distribution, 5.81% had undergone community-based maintenance therapy or clean needle distribution, and 19.13% had participated in peer education. A high proportion reported commercial or casual sexual partnerships within the last 3 months. A smaller proportion reported a history of male-to-male sexual behavior or intravenous drug use. The HIV and syphilis positive rates among male patients attending STD clinics were higher than those of other outpatients during the same period. Multivariate analysis indicated that non-local residency (odds ratio (OR) = 5.791), history of male-to-male sexual behavior (OR = 13.34), and a prior diagnosis of STD within the past year (OR = 4.106) were significant risk factors for HIV infection among STD clinic patients. Conducting HIV, syphilis, and hepatitis C testing in STD clinics can effectively identify more cases, improving screening efficiency and enabling early diagnosis and treatment for positive patients. Targeted preventive interventions are essential to enhance knowledge of HIV prevention and translate it into protective behaviors, reducing the occurrence of high-risk activities. This approach is critical to controlling the spread of AIDS and demonstrates significant representativeness in evaluating program outcomes.

Sexually transmitted diseases (STDs) refer to illnesses primarily transmitted through sexual contact, typically affecting the genital area.^[1]^ Over the past decade, the spectrum of STDs has expanded, with more than 20 identified sexually transmitted pathogens. In China, 8 STDs are prioritized for prevention and control, including human immunodeficiency virus (HIV)/ acquired immune deficiency syndrome (AIDS), syphilis, chancroid, lymphogranuloma venereum, chlamydial infections of the reproductive tract, genital warts, and genital herpes. Among these, HIV and syphilis often have insidious onsets with latent periods, and delayed detection may miss the optimal window for treatment.^[2]^ Male patients attending STD clinics are a key sentinel population for HIV surveillance,^[3]^ reflecting the risk of sexually transmitted HIV in the monitored area and serving as a significant avenue for detecting HIV and syphilis cases.^[4]^ From 2018 to 2021, a sentinel surveillance project focusing on male patients aged 15 years and older was implemented in the STD clinic of a monitoring hospital in Beilun District, Ningbo. This study analyzes sentinel surveillance data on AIDS knowledge awareness, AIDS-related behaviors, and HIV/syphilis/hepatitis C infection status, as well as factors influencing HIV infection, to evaluate the effectiveness of the surveillance program.

## 1. Objects and methods

### 1.1. Objects

From 2018 to 2021, male patients aged over 15 years who voluntarily and consecutively visited the dermatology and STD clinic, regardless of whether they had been diagnosed with an STD, were included in the surveillance program. Excluded from the study were individuals seeking reproductive medical advice, dermatology patients attending the clinic for skin diseases, and those recruited for various prevention and treatment programs. Each year, 400 cases were monitored.

### 1.2. Methods

A cross-sectional survey was conducted. For eligible participants who provided informed consent, one-on-one interviews were conducted by trained clinic physicians using a standardized questionnaire. The survey collected demographic characteristics, HIV-related behavioral characteristics, awareness of AIDS knowledge, and information about the acceptance of preventive interventions. After the questionnaire survey, 5 mL of venous blood was collected from each participant for HIV, syphilis, and hepatitis C virus (HCV) testing. Both hospital and district-level disease control centers performed dual sampling and double testing.

### 1.3. Definition of related indicators

AIDS knowledge awareness: The participant correctly answered 6 or more out of 8 questions related to AIDS prevention and treatment knowledge^[5]^; Relevant behavioral indicators: The number of participants who reported engaging in sexual activity, as the numerator, divided by the total number of participants, as the denominator; Intervention acceptance indicators: The number of participants who received any form of intervention service, as the numerator, divided by the total number of participants, as the denominator; Testing acceptance indicators: The number of participants who underwent testing, as the numerator, divided by the total number of participants, as the denominator; HIV positive detection rate: The number of antibody-positive individuals detected by both the sentinel hospital and the district disease control center (as the numerator), divided by the total number of individuals tested for HIV (as the denominator); and Syphilis positive detection rate: The number of individuals who tested positive for both syphilis-specific antibodies and nonspecific antibodies (as the numerator), divided by the total number of individuals tested for syphilis (as the denominator).

### 1.4. Laboratory testing

HIV antibody screening was performed using enzyme-linked immunosorbent assay (ELISA) kits (produced by Beijing Wantai Biological Pharmacy Enterprise Company Limited [Co. Ltd], Beijing, China). For those with initial positive results, a second ELISA test using a different domestic reagent (produced by Beijing Wantai Biological Pharmacy Enterprise Co. Ltd) was conducted. If the result was negative, the participant was considered negative and no further testing was done; if positive, the individual entered the confirmatory testing procedure. Confirmatory positive cases were sent to the Beilun District Disease Control Center for validation. Syphilis antibody screening was performed using the colloidal selenium method (produced by Beijing Wantai Biological Pharmacy Enterprise Co. Ltd). For initial positive results, a toludine red unheated serum test test kit (produced by Shanghai Kehua Bioengineering Co. Ltd, Shanghai, China) was used for confirmation. Confirmatory positive results were considered syphilis-positive. HCV testing was performed using 2 different ELISA kits for initial screening and confirmatory testing (produced by Shanghai Kehua Bioengineering Co. Ltd). If both tests were positive, the participant was considered HCV-positive.

### 1.5. Statistical analysis

Data were organized and analyzed using Excel 2010 (Microsoft Corporation Co., Ltd., Vancouver) and SPSS 18.0 (SPSS Inc., Chicago ) software. Categorical data were presented as frequencies and percentages, and univariate analysis was performed using the χ^2^ test. Variables with a *P*-value ≤ .10 in univariate analysis were included in multivariate logistic regression analysis.

## 2. Results

### 2.1. Demographic characteristics, AIDS knowledge awareness, behavioral intervention, and changes in high-risk behaviors

From 2018 to 2021, 1600 male patients attending STD clinics were surveyed. Of these, 1226 cases (76.69%) were aged 15 to 44, 307 cases (19.19%) were aged 45 to 64, and 67 cases (4.19%) were aged ≥65. A total of 490 cases (30.63%) were non-local residents, while 1110 cases (69.38%) were local residents, with a migrant-to-local ratio of 0.44:1. Married individuals accounted for 1160 cases (72.56%), while unmarried individuals accounted for 27.50%. Differences in marital status and place of residence across the years from 2018 to 2021 were statistically significant (*P* < .05), as shown in Table 1. The overall AIDS knowledge awareness rate from 2018 to 2021 was 76.18%. The percentage of individuals correctly answering 6 or more AIDS-related prevention and control questions increased annually: 53.25% in 2018, 73.50% in 2019, 86.50% in 2020, and 91.50% in 2021 (χ^2^ = 192.738, *P* = .000). Among the surveyed patients, 42.88% received condom promotion and distribution, 5.81% received community-based maintenance therapy or clean needle services, and 19.13% participated in peer education. The acceptance rates for these interventions varied significantly over the years (*P* ≤ .05), as shown in Table 1. Behavioral surveys revealed that 45.50% of participants had a history of commercial sexual activities within the past 3 months, mainly involving one (21.69%) or 2 (18.38%) female sex workers. Additionally, 44.50% reported casual sexual partners during the same period, primarily involving one (19.88%) or 2 (19.19%) casual partners. High-risk sexual behaviors were reported in nearly half of the participants. Furthermore, 1.25% of participants reported a history of injecting drugs, and 1.50% reported a history of male-to-male sexual behavior, with the latter showing a downward trend over the years, as shown in Table 3. The proportion of participants diagnosed with an STD in the past year decreased annually: 11.75% in 2018, 10.00% in 2019, 3.50% in 2020, and 3.00% in 2021 (χ^2^ = 36.441, *P* = .000). The most common STDs were syphilis (3.94%), gonorrhea (1.44%), and genital warts (1.13%), as shown in Table 1.

**Table 1:** Social demographic characteristics, awareness of AIDS knowledge, acceptance of interventions, and changes in high-risk behaviors among male patients attending STD clinics at Beilun District People’s Hospital (2018–2021) (%).

Factors	Variables	Categories	2018	2019	2020	2021	Total	χ^2^ value	*P* value
Demographic information	Place of residence	Local Province	240 (60.00)	245 (61.25)	326 (81.50)	299 (74.75)	1110 (69.38)	62.094	.000
		Other Provinces	160 (40.00)	155 (38.75)	74 (18.50)	101 (25.25)	490 (30.63)		
	Age group	15–44	311 (77.75)	313 (78.25)	294 (73.75)	308 (77.00)	1226 (76.69)	5.799	.466
		45–64	74 (18.50)	76 (19.00)	84 (21.00)	73 (18.25)	307 (19.19)		
		≥65	15 (3.75)	11 (2.75)	22 (5.50)	19 (4.75)	67 (4.19)		
	Marital status	Married	272 (68.00)	288 (72.00)	313 (78.25)	287 (71.75)	1160 (72.50)	10.859	.013
		Unmarried/Cohabiting/Divorced/Widowed	128 (32.00)	112 (28.00)	87 (21.75)	113 (28.25)	440 (27.50)		
Risk behavior factors	History of commercial sexual activities (last 3 mo)	Yes	190 (47.50)	237 (59.25)	106 (26.50)	195 (48.75)	728 (45.50)	91.078	.000
		No	210 (52.50)	163 (40.800)	294 (73.50)	205 (51.50)	872 (54.50)		
	Number of sexual partners (with sex workers)	1	83 (20.75)	94 (23.50)	42 (10.50)	75 (18.75)	294 (18.38)	8.635	.003
		2	85 (21.25)	124 (31.00)	44 (11.00)	94 (23.50)	347 (21.69)		
		3	17 (4.25)	17 (4.25)	18 (4.50)	17 (4.25)	69 (4.31)		
		4	5 (1.25)	2 (0.50)	2 (0.50)	1 (0.25)	10 (0.63)		
		Unclear	0 (0.00)	0 (0.00)	0 (0.00)	8 (2.00)	8 (0.50)		
	History of temporary sexual partners (last 3 mo)	Yes	187 (46.80)	198 (49.50)	154 (38.50)	173 (43.30)	712 (44.50)	10.953	.027
		No	213 (53.30)	202 (50.50)	246 (61.50)	227 (56.80)	888 (55.50)		
	Number of temporary sexual partners	1	96 (24.00)	65 (16.25)	94 (23.50)	63 (15.75)	318 (19.88)	9.797	.002
		2	71 (17.75)	112 (28.00)	43 (10.75)	81 (20.25)	307 (19.19)		
		3	12 (3.00)	19 (4.75)	16 (4.00)	18 (4.50)	65 (4.06)		
		4–5	8 (2.00)	2 (0.50)	1 (0.25)	2 (0.50)	13 (0.81)		
		Unclear	0 (0.00)	0 (0.00)	0 (0.00)	9 (2.25)	9 (0.56)		
	Injection drug use	Yes	4 (1.00)	0 (0.00)	12 (3.00)	4 (1.00)	20 (1.30)	15.392	.004
		No	396 (99.00)	400 (100.00)	388 (97.00)	396 (99.00)	1580 (98.80)		
	History of anal sexual intercourse with males	Yes	20 (5.00)	2 (0.50)	1 (0.30)	1 (0.30)	24 (1.50)	44.332	.000
		No	380 (95.00)	398 (95.50)	399 (99.80)	399 (99.80)	1576 (98.50)		
	Diagnosis of STD in the last year	Yes	47 (11.75)	40 (10.00)	14 (3.50)	12 (3.00)	113 (7.06)	36.441	.000
		No	353 (88.25)	360 (90.00)	386 (96.50)	388 (97.00)	1487 (92.94)		
	Type of STD previously contracted	Gonorrhea	12 (3.00)	4 (1.00)	1 (0.25)	1 (0.25)	18 (1.13)	31.348	.000
		Syphilis	17 (4.25)	25 (6.25)	11 (2.75)	10 (2.50)	63 (3.94)		
		Chlamydia infection	2 (0.50)	1 (0.25)	0 (0.00)	0 (0.00)	3 (0.19)		
		Genital warts	13 (3.25)	8 (2.00)	2 (0.50)	0 (0.00)	23 (1.44)		
		Genital herpes	3 (0.75)	2 (0.50)	0 (0.00)	1 (0.25)	6 (0.38)		
Protective behavior factors	Awareness of AIDS knowledge	Yes	213 (53.25)	294 (73.50)	346 (86.50)	366 (91.50)	1219 (76.18)	192.738	.000
		No	187 (46.80)	106 (26.50)	54 (13.50)	34 (8.50)	381 (23.80)		
	Condom promotion and distribution	Yes	228 (57.00)	172 (43.00)	218 (54.50)	68 (17.00)	686 (42.88)	164.000	.000
		No	172 (43.00)	228 (57.00)	182 (45.50)	332 (83.00)	914 (57.12)		
	Community-based methadone maintenance/Clean needle programs	Yes	4 (1.00)	52 (13.00)	37 (9.25)	0 (0.00)	93 (5.81)	87.985	.000
		No	396 (99.00)	348 (87.00)	363 (90.75)	400 (100.00)	1507 (94.19)		
	Peer education	Yes	102 (25.50)	110 (27.50)	40 (10.00)	54 (13.50)	306 (19.13)	58.365	.000
		No	298 (74.50)	290 (72.50)	360 (90.00)	346 (86.50)	1294 (80.87)		

AIDS = acquired immune deficiency syndrome, HIV = human immunodeficiency virus, STD = sexually transmitted disease.

### 2.2. Serological testing results

The HIV detection rate among male patients was 6.88‰, significantly higher than the 0.02‰ detection rate among general outpatients during the same period (χ^2^ = 2288.01, *P* = .000). Among STD clinic patients, 2 HIV cases were detected in 2018, and 3 cases were detected each year from 2019 to 2021, showing a slight upward trend. In contrast, the HIV detection rate among general outpatients remained stable. The syphilis detection rate among male patients was 66.88‰, significantly higher than the 0.30‰ detection rate among general outpatients (χ^2^ = 19,212.687, *P* = .000). The syphilis detection rate among STD clinic patients showed a decreasing trend from 2018 to 2021 (χ^2^ = 1.504, *P* = .681). The HCV detection rate among male patients was 1.75‰, slightly higher than the 1.43‰ rate among general outpatients, with statistically significant difference (χ^2^ = 11.503, *P* = .001). However, the HCV positive rate among general outpatients showed an increasing trend (χ^2^ = 92.269, *P* = .000), as shown in Table 2.

**Table 2:** HIV, syphilis, and hepatitis C positive detection rates (‰) for male patients in the STD clinic from 2018 to 2021 compared with concurrent outpatient clinic patients.

Year		2018	2019	2020	2021	Total	χ^2^ value	*P* value
HIV detection								
STD clinic male patients	Number of visits	400	400	400	400	1600	4.314	.229
	Positive detection (rate)	2 (5.00)*	3 (7.50)*	3 (7.50)*	3 (7.50)*	11 (6.88)*		
Outpatient clinic patients	Number of visits	380,648	391,791	298,841	366,366	1,437,646	3.181	.365
	Positive detection (rate)	6 (0.02)	8 (0.02)	7 (0.02)	11 (0.02)	32 (0.02)		
Total	Number of visits	381,048	392,191	299,241	366,766	1,439,246	3.631	.304
	Positive detection (rate)	8 (0.02)	11 (0.03)	10 (0.03)	14 (0.04)	43 (0.11)		
Syphilis detection								
STD clinic male patients	Number of visits	400	400	400	400	1600	1.504	.681
	Positive detection (rate)	32 (80.00)*	25 (62.50)*	24 (60.00)*	26 (65.00)*	107 (66.90)*		
Outpatient clinic patients	Number of visits	380,648	391,791	299,241	366,366	1,437,646	4.538	.209
	Positive detection (rate)	124 (0.33)	113 (0.29)	92 (0.31)	90 (0.25)	419 (0.29)		
Total	Number of visits	381,048	392,191	299,241	366,766	1,439,246	5.079	.166
	Positive detection (rate)	156 (0.41)	138 (0.35)	116 (0.39)	116 (0.32)	526 (0.37)		
Hepatitis C detection								
STD clinic male patients	Number of visits	400	400	400	400	1600	4.314	.229
	Positive detection (rate)	3 (0.75)	2 (0.5)	0 (0.00)	2 (0.5)*	7 (1.75)		
Outpatient clinic patients	Number of visits	5178	8633	10,144	12,366	36,321	92.269	.000
	Positive detection (rate)	53 (1.02)	80 (0.92)	104 (1.03)	283 (2.29)	520 (1.43)		
Total	Number of visits	5578	9033	10,544	12,766	37,921	94.001	.000
	Positive detection (rate)	56 (1.00)	82 (0.91)	104 (0.99)	285 (2.23)	527 (1.39)		

HIV = human immunodeficiency virus, STD = sexually transmitted disease.

* Compared to outpatient clinic patients, the positive detection rates for male patients in the STD clinic showed statistical differences. *P* < .05.

### 2.3. Factors associated with HIV infection

Univariate logistic analysis identified several factors associated with HIV infection among STD clinic patients, including residency, history of male-to-male sexual behavior, diagnosis of an STD within the past year, and participation in peer education. Variables with *P* < .10 in univariate analysis were included in multivariate logistic regression analysis. The results showed that non-local residency (odds ratio (OR) = 5.791), a history of male-to-male sexual behavior (OR = 13.34), and a diagnosis of an STD within the past year (OR = 4.106) were significant risk factors for HIV infection. A history of history of male-to-male sexual behavior posed the highest risk (see Table 3).

**Table 3:** Analysis of factors associated with HIV infection among male patients at STD sentinel clinics in Beilun District People’s Hospital (2018–2021).

Variable	Sample size	HIV cases	HIV infection rate (%)	Univariate analysis		Multivariate analysis	
				OR value (95% CI)	*P* value	aOR value (95% CI)	*P* value
Monitoring year							
2018	400	2	0.50	/	.965		
2019	400	3	0.80	0.665 (0.111, 4.001)	.656		
2020	400	3	0.80	1 (0.201, 4.985)	1		
2021	400	3	0.80	1 (0.201, 4.985)	1		
Place of residence							
Local province	1110	3	0.30	/	0		
Other provinces	490	8	1.60	6.124 (1.618, 23.185)	.008	5.791 (1.509, 22.219)	.01
Age group (yr)							
15–44	1226	8	0.70	/	.836		
45–64	307	3	1.00	10610675.486 (0, /)	.997	–	–
≥65	67	0	0.00	15942190.882 (0, /)	.997	–	–
Marital status							
Married	1160	6	0.50	/	0		
Single/Cohabiting/Divorced/Widowed	440	5	1.10	2.211 (0.671, 7.281)	.192		–
Awareness of HIV knowledge							
No	381	3	0.80	/	0		
Yes	1219	8	0.70	0.832 (0.22, 3.153)	.787		
History of sexual activities with sex workers in the past 3 mo							
No	872	5	0.60	/	0		
Yes	728	6	0.80	1.441 (0.438, 4.741)	.548	–	–
History of sexual activities with temporary sexual partners in the past 3 mo							
No	888	4	0.50	/	0		
Yes	712	7	1.00	2.194 (0.64, 7.526)	.211	–	–
History of drug injection							
No	1580	11	0.70	/	0		
Yes	20	0	0.00	0	.999		
History of anal intercourse with men							
No	1576	9	0.60	/	0		
Yes	24	2	8.30	15.828 (3.231, 77.533)	.001	13.34 (2.454, 72.519)	.003
Diagnosed with STD in the last year					0.037		–
No	1487	8	0.50	/	0		
Yes	113	3	2.70	5.042 (1.319, 19.274)	.018	4.106 (1.008, 16.72)	.049
Condom promotion and distribution							
No	914	7	0.80	/	0		
Yes	686	4	0.60	1.316 (0.384, 4.513)	.662		
Community-based maintenance therapy/Clean needle programs							
No	1507	11	0.70	/	0		
Yes	93	0	0.00	11878482.263 (0, /)	.997		
Peer education							
No	1294	6	0.50	/	0		
Yes	306	5	1.60	0.28 (0.085, 0.925)	.037	0.487 (0.139, 1.708)	.261
Most recent HIV test result							
Negative	1429	8	0.60	/	0		
Positive	171	3	1.80	3.172 (0.833, 12.071)	.09		
Most recent syphilis test result							
Negative	1493	9	0.60	/	0		
Positive	107	2	1.90	3.141 (0.670, 14.722)	.147		
Most recent hepatitis C test result							
Negative	1589	11	0.70	/	0		
Positive	11	0	0.00	0 (0, /)	.999		

CI = confidence interval, HIV = human immunodeficiency virus, OR = odds ratio, STD = sexually transmitted disease.

## 3. Discussion

The HIV sentinel surveillance system involves the regular and systematic collection and analysis of HIV-related data and behavioral characteristics among specific populations. This process aims to assess the epidemic trends of HIV/AIDS, identify high-risk behavioral patterns, and provide a scientific basis for formulating HIV prevention and control strategies. Healthcare institutions play a vital role in STD testing, treatment, and support services.^[6]^ Male patients attending STD clinics are a key sentinel population for HIV surveillance. A surveillance study conducted from 2018 to 2021 at a sentinel hospital in Beilun District, Ningbo in male patients attending STD clinics through questionnaires and tests for HIV, syphilis, and hepatitis C, revealed that STD clinic male attendees were primarily young and middle-aged individuals (aged 15–44), a sexually active demographic that is a key target group for AIDS and STD prevention. This aligns with findings from related studies.^[7,8]^ Most participants were married and local residents, indicating that married local populations voluntarily participate in STD testing more than other groups. From 2018 to 2021, the HIV detection rate among STD clinic male attendees was 6.88‰, significantly higher than the 0.02‰ detection rate among general outpatients during the same period. Similarly, the syphilis detection rate among STD clinic attendees (66.90‰) was markedly higher than that of general outpatients (0.29‰). These results highlight the cost-effectiveness of conducting HIV and syphilis testing in STD clinics to identify positive cases early, which is consistent with other studies.^[9]^ In recent years, cases of HCV transmitted through sexual contact have increased, with the co-infection rate of HIV and HCV in China reported at 8%. In this study, the HCV detection rate among male attendees of STD clinics was 1.75‰, higher than the detection rate among general outpatients (1.43‰). This finding underscores the importance of integrating HCV testing into STD clinic services, especially since HCV shares similar transmission routes with HIV. Early detection and treatment of HCV can reduce liver damage and lower HIV-related complications.^[10]^ The HIV, syphilis, and HCV detection rates among male attendees of the sentinel hospital’s STD clinic were lower than those reported in other regions such as Tianjin and Shanxi.^[11,12]^ These regional differences may be attributed to variations in economic and cultural factors, population behavioral characteristics, and medical intervention measures.

Between 2018 and 2021, the AIDS knowledge awareness rate increased from 53.25 to 91.50%, with an overall awareness rate of 76.18%, higher than those reported in other studies.^[13,14]^ This upward trend reflects significant improvements in public awareness due to years of AIDS education efforts. However, awareness of specific topics – such as the inability to identify AIDS by appearance (65.56%), the fact that mosquito bites do not transmit AIDS (63.75%), and the lack of risk from eating with AIDS patients (66.75%) – remains low. These findings align with other studies^[15,16]^ and suggest the need for targeted, straightforward educational efforts in STD clinics. Methods such as brochures, posters, and digital platforms in clinic waiting areas could improve knowledge among key populations.^[17,18]^ Regarding behavioral interventions, the survey revealed that 42.88% of male attendees had received condom promotion and distribution, 19.13% participated in peer education, and only 5.81% accessed community-based maintenance therapy or clean needle services. This suggests a need to enhance the availability of maintenance therapy and clean needles in communities to reduce the risk of disease transmission among drug users.^[19]^

Behavioral monitoring from 2018 to 2021 indicated that 45.50% of participants had engaged in commercial sexual activities within the past 3 months, with the lowest incidence in 2020 likely due to the COVID-19 pandemic and related closures of entertainment venues. The incidence increased again in 2021 following adjustments to pandemic control policies.^[20]^ Similarly, the proportion of participants with casual sexual partners exceeded 40% in most years, consistent with trends in commercial sexual activities.^[21]^ These behaviors act as a “bridge” for STD transmission from high-risk groups to the general population.^[22]^ Additionally, a proportion of male attendees reported male-to-male sexual behavior each year, emphasizing the need for health education targeting male sexual behavior to prevent STD spread.The proportion of participants with a history of STDs in the past year decreased from 11.75% in 2018 to 3.00% in 2021. This decline may be linked to COVID-19 pandemic measures, which reduced travel, closed entertainment venues, and lowered clinic attendance.^[20,23]^

Multivariate logistic regression analysis indicated that non-local residency is a significant factor associated with HIV infection among STD clinic attendees, as migrant populations often experience unstable living conditions and higher risks of high-risk behaviors.^[19]^ A history of history of male-to-male sexual behavior posed the highest risk (OR = 13.34), while an STD diagnosis in the past year was also a risk factor. These findings align with multiple studies, showing that risky sexual behaviors, particularly male-to-male sexual activities, are critical risk factors for HIV infection.^[24]^ Notably, although HIV knowledge awareness rates have increased, multivariate analysis did not identify awareness as a protective factor. Peer education showed protective effects in univariate analysis but was excluded in multivariate analysis. These findings suggest that knowledge alone is insufficient; interventions must focus on promoting behavioral changes among high-risk groups to encourage protective measures.^[19]^

This study cannot conduct a time trend analysis of HIV, syphilis, and hepatitis C positive rates because of only four-year data. The HIV, syphilis, and hepatitis C testing results of male patients in the STD clinic were compared with those of patients in the same period in the clinic of this sentinel unit, the testing efficiency cannot be clearly demonstrated by indicators such as OR value. However, HIV, syphilis, and HCV testing among male attendees of STD clinics facilitates early diagnosis and treatment, demonstrating significant clinical value. Tailored prevention and intervention measures should be developed for specific populations, such as migrants, to transform HIV knowledge into protective actions, reduce risky behaviors, and effectively control the AIDS epidemic.

## Author contributions

**Data curation**: Libo Zhang, Yingbin Wang, Ludan Xu.

**Formal analysis**: Ludan Xu.

**Supervision**: Yaner Yu.

**Writing – original draft**: Yajun Ding, Dongxian Ye.

**Writing – review & editing**: Chunxia Xu.
